# Balance confidence classification in people with a lower limb amputation using six minute walk test smartphone sensor signals

**DOI:** 10.1371/journal.pdig.0000570

**Published:** 2024-08-26

**Authors:** Pascale Juneau, Natalie Baddour, Helena Burger, Edward D. Lemaire

**Affiliations:** 1 Ottawa Hospital Research Institute, Ottawa, Canada; 2 Department of Mechanical Engineering, University of Ottawa, Ottawa, Canada; 3 University Rehabilitation Institute, University of Ljubljana, Ljubljana, Slovenia; 4 Faculty of Medicine, University of Ljubljana, Ljubljana, Slovenia; 5 Faculty of Medicine, University of Ottawa, Ottawa, Canada; University of Ulm, GERMANY

## Abstract

The activities-specific balance confidence scale (ABC) assesses balance confidence during common activities. While low balance confidence can result in activity avoidance, excess confidence can increase fall risk. People with lower limb amputations can present with inconsistent gait, adversely affecting their balance confidence. Previous research demonstrated that clinical outcomes in this population (e.g., stride parameters, fall risk) can be determined from smartphone signals collected during walk tests, but this has not been evaluated for balance confidence. Fifty-eight (58) individuals with lower limb amputation completed a six-minute walk test (6MWT) while a smartphone at the posterior pelvis was used for signal collection. Participant ABC scores were categorized as low confidence or high confidence. A random forest classified ABC groups using features from each step, calculated from smartphone signals. The random forest correctly classified the confidence level of 47 of 58 participants (accuracy 81.0%, sensitivity 63.2%, specificity 89.7%). This research demonstrated that smartphone signal data can classify people with lower limb amputations into balance confidence groups after completing a 6MWT. Integration of this model into the TOHRC Walk Test app would provide balance confidence classification, in addition to previously demonstrated clinical outcomes, after completing a single assessment and could inform individualized rehabilitation programs to improve confidence and prevent activity avoidance.

## Author summary

People with lower limb amputations have a high risk of falling and typically report low balance confidence levels. Low balance confidence can prevent people from engaging in physical and social activities. Previous research demonstrated that smartphone signals collected during walking can be used with machine learning to predict clinical outcomes in these individuals (e.g., stride parameters, fall risk). In this research, we investigated if balance confidence scores, categorized into high confidence and low confidence groups, could be classified. We demonstrate that smartphone signal data can stratify people with a lower limb amputation into high and low balance confidence groups after they complete a 6-minute walk test, correctly classifying over 80% of participants. The paper also discusses the relationship between balance confidence and ability, and how identifying those with mismatched balance ability and confidence scores (i.e., high confidence, but poor balance ability) could improve outcomes.

## 1 Introduction

Balance confidence has been defined as “an individual’s confidence in their ability to maintain their balance while performing various activities” [1]. Balance confidence can have a broad effect on quality of life; including, decreased health status, functional decline, depression and anxiety, and activity avoidance [2–5]. Clinically, the activities-specific balance confidence scale (ABC) has become a common tool to assess confidence when moving within a person’s chosen environment.

ABC is a 16-item questionnaire that evaluates a person’s confidence in completing different activities that may challenge their balance; such as, walking indoors and out-doors on different surfaces, using stairs, and reaching for items overhead under different conditions (Table 1) [6]. Participants are asked to rate their confidence performing each of these activities without becoming unsteady, on a scale of 0% (no confidence) to 100% (complete confidence). If the person normally uses a mobility aid or other support while performing these activities, they are encouraged to complete the questionnaire as if they had access to their aids while performing the activity. The ABC scale is also used to evaluate various at-risk populations and is considered a reliable balance confidence measure [7–10] for people with lower limb amputations [10]. The final score is an average across each activity out of 100%. A score of 80% or greater is considered high confidence and can indicate higher physical function and decreased fall risk [11].

**Table 1:** Activities-Specific Balance Confidence (ABC) Scale.

How confident are you that you will not lose your balance or become unsteady when. . .
1. Walking around the house
2. Walking up or down stairs
3. Bending over to pick up a slipper from the front of a closet floor
4. Reaching for a small can off a shelf at eye level
5. Standing on tiptoes and reaching for something above his/her head
6. Standing on a chair to reach for something
7. Sweeping the floor
8. Walking outside the house to a car parked in the driveway
9. Getting into or out of a car
10. Walking across a parking lot to the mall
11. Walking up or down a ramp
12. Walking in a crowded mall where people rapidly walk past
13. Being bumped into people as they walk through the mall
14. Stepping on to or off an escalator while holding onto a railing
15. Stepping onto or off an escalator while holding onto parcels (so that you are not able to hold the railing)
16. Walking outside on icy sidewalks

People with lower limb amputations present with increased gait variability and instability that put them at elevated risk of falls during all stages of rehabilitation [12]. They are at a greater risk of falls compared to age-matched able-bodied populations [13]. These individuals report low balance confidence scores, with some research reporting average ABC scores between 62.8 and 63.8% [14,15,16]. People who had a vascular amputation reported lower average scores (50.6–54.1%) compared to those whose amputation was due to a different cause [7,14]. Asano et al. [15] demonstrated that an ABC score of <80% significantly decreased quality of life scores in people with lower limb amputations and indicated that the ability to move about one’s environment safely was moderately related to their perceived quality of life.

While high confidence can indicate higher functional ability in older adults, greater balance ability in people with lower limb amputations can increase fall risk. Moreover, high balance confidence imposes an elevated risk for this population if this confidence is mismatched with the individual’s ability. One study reported that, for people with a lower limb amputation, risk of falling was 3.7 times greater if balance ability was low, but confidence scores were high [17]. Steinberg et al. [12] concluded that excess confidence in balance and walking abilities, less caution during stair walking, and higher gait variability increased fall risk in people with lower limb amputation. Early identification of those at elevated risk of falls, particularly those with mismatched balance confidence and ability is important to ensure that preventative strategies, such as education, exercise, or monitoring, can be implemented.

In previous research, Juneau et al. [18] demonstrated that important clinical outcomes for people with lower limb amputation could be calculated using smartphone sensor signals collected during a 6-minute walk test (6MWT). Stride parameters (e.g., step time, stride time) and step-based features were used to train a random forest to classify fall risk. Participants were identified as “fall risk” if they had at least one self-reported fall in the six months prior to data collection, consistent with clinical assessments. Stride parameter calculation and fall risk classification were equivalent to results achieved when manually-labelled foot strikes were used, indicating that automated foot strikes and smartphone signals were a clinically viable approach. The 6MWT is a commonly used movement assessment that evaluates functional capacity in several populations, including people with lower limb amputations. Despite not being designed as a fall risk measure, the research in [18] illustrated that additional clinical information could be extracted from the 6MWT task.

Understanding a person’s perception of their balance abilities is an essential part of the rehabilitation process in recovery from a fall and confidence levels can affect behaviours that could lead to future falls. In this paper, we are interested in classifying an ABC score from 6MWT data. To achieve this task, we must first choose between using a classification or regression approach. The choice depends on the specific goals, categorization, or prediction. In this research, a random forest algorithm was selected. Random forests are machine learning algorithms that consist of multiple decision tree classifiers. Each decision tree in the ensemble provides an individual classification result. The final classification is the class with the most predictions across all trees. Our goal is to categorize balance confidence, thereby enhancing the information a clinician can obtain after a 6MWT. Furthermore, the relationship between the 6MWT and ABC score is likely nonlinear, which lends further support to using a classification approach.

Given the relationship between balance ability and confidence level, classification of balance confidence in people with lower limb amputations could allow for a more individualised rehabilitation approach, especially when combined with clinical measures of balance ability. In this paper, we investigated if low and high balance confidence groups could be classified using 6MWT smartphone signal data and automated foot strike detection collected from people with lower limb amputations. Classification results were analyzed alongside self-reported fall history to investigate the relationship between self-reported confidence and reported balance history.

## 2 Materials and methods

### 2.1 Recruitment and participants

Individuals with transtibial, transfemoral, and bilateral lower limb amputations were recruited from the outpatient clinic at the University Rehabilitation Institute (Ljubljana, Slovenia). All participants provided informed consent. This research was approved by the Ethic Committee of the University Rehabilitation Institute, Slovenia (# 46/2018) and re-approved for 30 additional participants (# 27/2019).

**Fig 1:**
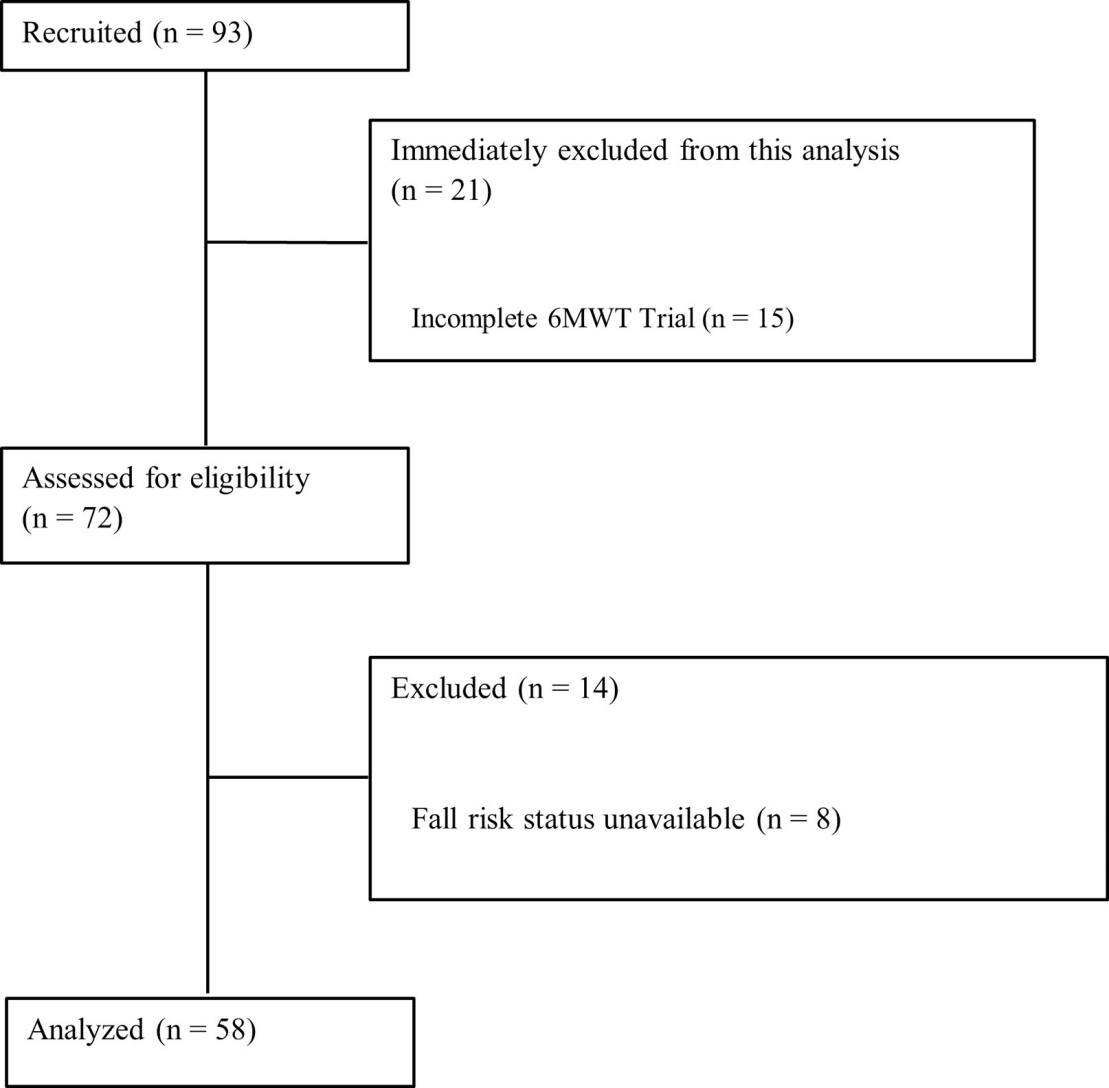
Inclusion/Exclusion Flowchart.

Participants provided information regarding their amputation, falls in the past six months (i.e., fall risk group), and ABC score (Table 2). An ABC score of 80% or greater was considered high confidence [11]. The inclusion criteria were: transtibial amputation or higher; ability to walk with single cane, two crutches, or without any walking aids; minimum of six months post-amputation; had a functional prosthesis; no injuries on the residual limb; was willing to participate and consented to participate. 93 people with lower limb amputations were recruited based on these criteria (Fig 1). Only participants who completed a full 6MWT trial and completed an ABC questionnaire were included in this analysis. Trials were excluded due to incomplete trial (15), fall risk status unavailable (8), ABC score unavailable (6), cell phone placed at the side of the hip instead of lower back (5), and use of a non-rolling walker (1).

**Table 2:** Participant demographics.

Age (years)	62.3 ± 12.8 (19–90)
Male	49 (84.5%)
Female	9 (15.5%)
Faller	19 (32.8%)
Non faller	36 (62.0%)
Unavailable	3 (5.2%)
Transtibial	51 (87.9%)
Transfemoral	4 (6.9%)
Bilateral (Transtibial)	3 (5.2%)
Time since amputation (years)	18.5 ± 18.8 (<1–65)
ABC score (%)	62.8 ± 28.5 (0–100)
High confidence (≥80%)	19 (32.8%)
Low confidence (<80%)	39 (67.2%)

Note: Data are presented as mean ± SD (range) or number (percentage).

### 2.2 Data collection

Each participant completed one 6MWT trial along a 20 m hallway with an Android smartphone placed in a belt at the lower back. Participants were video recorded during their walk test. The TOHRC Walk Test app collected smartphone accelerometer, gyroscope, and orientation data at 50 Hz [19]. Raw smartphone signal data and timestamps for each participant trial were imported into MATLAB (MathWorks) 2020b [20] for preprocessing. Since smartphone signals have a variable sampling rate, each signal was linearly interpolated to a fixed 50 Hz for a total of 18,049 data points per signal per participant over the 6MWT. Signals were then filtered with a fourth-order zero-lag Butterworth low pass filter with a 4Hz cut-off frequency [21].

### 2.3 Feature calculation and classification model

Automated foot strikes were used to calculate step-based features. The automated step detection model was described in [22]. Predicted foot strike labels were post-processed in MATLAB (MathWorks) 2020b to correct for model prediction errors, including extra foot strike predictions and missed steps. Typically, a single foot strike event would correspond with a peak in the anterior-posterior (AP) acceleration signal. Extra predictions were defined as instances where two or more consecutive predicted foot strikes were present on the same AP signal, usually immediately before or after the peak in the signal. The timepoints corresponding to these consecutive foot strike predictions were identified. The foot strike event corresponding to the AP acceleration peak was selected and all other predictions in this period were removed.

To identify missed steps, a method similar to that employed by Capela et al. [19] was applied. The duration between each step was recorded. Periods where the duration between two consecutive steps was at least 1.5 times greater than the previous pair of steps were identified. An adaptive locking period specific to each participant’s trial was applied and searched for potential missed steps. Within the adaptive locking period, the AP acceleration peak was identified, and a foot strike was inserted at this timestamp. Final cleaned predictions were used for feature calculations.

62 features were extracted for each step (Table 3). Then the minimum, maximum, mean, and standard deviation were calculated over all steps for a total of 248 features (62 features multiplied by 4 statistics) Table 3. These 248 features were used as input for the random forest model to classify balance confidence groups. The random forest was built and run in Weka 3.8 (Developer University of Waikato) [23]. Correlation-based feature selection (CFS) was used to reduce dimensionality. Participant level leave-one-out cross-validation was used to evaluate classification model performance.

**Table 3:** Feature list for fall risk classification. AP = anterior-posterior; ML = medio-lateral; RMS = root-mean square; FFT = fast Fourier transform; REOH = ratio of even/odd harmonic frequencies.

Temporal	Descriptive Statistics	Frequency Domain Features
Cadence	Minimum ML	Quartile FFT ML
Step time right	Minimum AP	Quartile FFT AP
Step time left	Minimum Vert	Quartile FFT Vert
Stride time	Maximum ML	Quartile FFT Tilt
Symmetry index^a^	Maximum AP	Quartile FFT Rotation
	Maximum Vert	Quartile FFT Obliquity
	Mean ML	Maximum FFT ML
	Mean AP	Maximum FFT AP
	Mean Vert	Maximum FFT Vert
	Mean Tilt	Maximum FFT Tilt
	Mean Rotation	Maximum FFT Rotation
	Mean Obliquity	Maximum FFT Obliquity
	Range Tilt	Standard Deviation FFT ML
	Range Rotation	Standard Deviation FFT AP
	Range Obliquity	Standard Deviation FFT Vert
	Standard Deviation ML	Standard Deviation FFT Tilt
	Standard Deviation AP	Standard Deviation FFT Rotation
	Standard Deviation Vert	Standard Deviation FFT Obliquity
	Standard Deviation Tilt	Peak Distinction FFT ML
	Standard Deviation Rotation	Peak Distinction FFT AP
	Standard Deviation Obliquity	Peak Distinction FFT Vert
	RMS ML	Peak Distinction FFT Tilt
	RMS AP	Peak Distinction FFT Rotation
	RMS Vert	Peak Distinction FFT Obliquity
	RMS Tilt	REOH ML
	RMS Rotation	REOH AP
	RMS Obliquity	REOH Vert
		REOH Tilt
		REOH Rotation
		REOH Obliquity

^a^Symmetry index: symmetry in right and left limb step times [24]

## 3 Results

For this study, 58 participants were suitable for analysis, 36 non-fallers and 19 fallers (Table 2). Participants were majority male (84.5%), and most participants (male and female) had a transtibial amputation (87.9%). Final model performance was based on 58 individually trained models after the leave-one-out cross-validation. CFS reduced the dataset to 18 step-based features. Self-reported fall history was not used as a feature for the ABC algorithm.

The average ABC score was 62.8%, with only 19 of 58 (32.8%) participants reporting a final score of 80% or greater. Both fallers and non-fallers were distributed across both low confidence and high confidence groups (Table 4), though a greater proportion of both groups were categorized as “low confidence”.

**Table 4:** Balance confidence from ABC questionnaire versus fall risk.

	Faller	Non-faller
High Confidence	5	13
Low Confidence	14	23

Table 5 details the average responses of individual ABC questions. Scoring across individual ABC questions was not consistent, with some activities resulting in lower average scores than others. Questions 15 and 16 reported the lowest average scores, with the second fewest (stepping onto or off an escalator while holding onto parcels) and fewest (walking outside on icy sidewalks) responses of 100% confidence. “Standing on a chair to reach for something” had the greatest number of 0% confidence responses. The criterion that received the highest average score and the greatest number of 100% confidence responses was “reaching for a small can off a shelf at eye level”.

**Table 5:** ABC Results.

Question	Average Score (%)	# of 0% confidence	# of 100% confidence
1	88	0	32
2	76	0	21
3	79	3	24
4	91	0	37
5	56	13	13
6	51	19	17
7	55	13	17
8	87	0	33
9	87	0	32
10	85	1	31
11	64	3	14
12	74	2	21
13	50	14	14
14	54	13	15
15	46	18	10
16	37	16	6

Table 6 displays the confusion matrix for balance confidence classification. Balance confidence classification accuracy was 81%, sensitivity 63.2%, specificity 89.7%, and precision 75.0%.

**Table 6:** Balance confidence classification.

		Predicted Class	
		High Confidence	Low Confidence
True Class	High Confidence	12	7
	Low Confidence	4	35

## 4 Discussion

This research demonstrated that balance confidence scores of people with lower limb amputations could be classified using smartphone 6MWT data and automated foot strikes when balance confidence was categorised into high and low confidence groups. Balance confidence classification was better for those in the low confidence group, which is the group that needs identification for possible interventions. Approximately two thirds (67.8%) of participants included in this analysis were low confidence, with an average score of 62.8%, which is consistent with the literature.

81% accuracy would provide correct classification for most people completing a 6MWT, thereby providing balance confidence knowledge that would not have been available without completing the questionnaire. 89.7% specificity and 75% precision showed that few people with low balance confidence were misclassified, so efforts to further investigate potential balance issues for a patient classified to "low confidence" would not be a large drain on resources due to unnecessary investigations (e.g., completing an ABC questionnaire to verify results, etc.).

Fear of falling can affect a person’s balance confidence, regardless if the fall resulted in an injury [25]. Identifying people with low balance confidence is essential, especially for those who may be restricting their social or physical activity participation due to decreased confidence. Clinicians could tailor treatment by providing strategies to improve balance and prevent future falls, but also to increase confidence and promote participation in social and physical activities. Miller et al [14] reported that limitations in activities of daily living, depression, and fear of falling were statistically related to balance confidence, highlighting the importance of addressing social and mental health factors in addition to functional ability for people with lower limb amputations.

It is important to acknowledge that the cut off value determined by Myers et al. [11] was developed for older adults. This study included data from a majority male population (84.5%) with mostly single transtibial amputations (87.9%). This reflects the distribution of males and females with lower limb amputations. However, the high percentage of male participants may limit the ability of this classification model to be applied to women with lower limb amputations, and future work should include a greater percentage of female participants. People with transtibial amputations are at a higher risk of falling during the post-operative period, while people with transfemoral and bilateral amputations are more likely to fall once they have completed their rehabilitation [26]. Including a greater number of people with transfemoral and bilateral amputations would improve the model’s generalizability and possibly allow for sub-group analysis. Additionally, this research only included people who were able to complete the full 6MWT. Excluding participants who were unable or unwilling to complete the full trial potentially excluded people with reduced mobility, less active individuals, or people with poorer balance.

For people with lower limb amputations, when high confidence is not reflected by the person’s physical ability, fall risk is greatly increased [12,17]. This is possibly because these individuals may feel confident enough to engage in more activities. For example, in [12] less caution during stair walking was identified as a risk factor for falls in people with lower limb amputation. Indeed, five people categorised in the high confidence group were self-reported fallers, one of whom reported an ABC score of 100% (Table 4). Interestingly, 23 of the low confidence participants had not fallen in the previous 6 months, indicating that there may not have been a direct relationship between previous falls and balance confidence score. The mechanisms that contribute to falls are diverse and balance confidence alone is not enough to identify individuals who are at risk of falls in this population.

Previous research [18] demonstrated that signal data collected from a smartphone during a 6MWT could be used to calculate stride parameters and provide preliminary fall risk information in people with lower limb amputations. Combining balance confidence scores with a measure of functional ability (i.e., 6MWT) might provide a clearer picture of an individual’s fall risk and would allow for clinicians to create a more individualized treatment program. It is important to note that this model does not replace the value of completing patient interviews. For example, the ABC questionnaire can identify specific activities of interest, like walking outside on icy sidewalks, that could be targeted for balance training. However, for use at the point of patient contact, these models could be integrated into the TOHRC Walk Test App for immediate reporting after completing a walk test.

## 5 Conclusions

This research showed that balance confidence can be classified in people with lower limb amputations using smartphone signals collected during a 6MWT. While balance ability and balance confidence may not always correspond, both affect an individual’s risk of future falls and quality of life. Identifying participants who have mismatched balance confidence and functional ability is not only essential to providing the appropriate resources and rehabilitation to prevent future falls, but also to prevent social isolation due to fear of falling. Incorporating balance confidence, in addition to stride parameter calculation and fall risk analysis, into the TOHRC Walk Test can enhance the 6MWT and provide additional information after completing a single assessment.
